# Determinants of Glucose Tolerance in a Population Without Overt Diabetes: The Role of β-Cell Glucose Sensitivity, Insulin Sensitivity, and Insulin Clearance

**DOI:** 10.3390/metabo16040218

**Published:** 2026-03-26

**Authors:** Beatrice Marelli, Andrea Foppiani, Federica Sileo, Giorgia Pozzi, Silvia Gallosti, Chiara Cappellini, Andrea Mari, Simona Bertoli, Alberto Battezzati

**Affiliations:** 1 International Center for the Assessment of Nutritional Status and the Development of Dietary Intervention Strategies (ICANS-DIS), Department of Food, Environmental and Nutritional Sciences (DeFENS), University of Milan, 20133 Milan, Italy; beatrice.marelli@studenti.unimi.it (B.M.);; 2 IRCCS Istituto Auxologico Italiano, Clinical Nutrition Unit, Department of Endocrine and Metabolic Medicine, 20100 Milan, Italy; 3 Institute of Neuroscience, National Research Council, 35127 Padova, Italy; 4 IRCCS Istituto Auxologico Italiano, Obesity Unit and Laboratory of Nutrition and Obesity Research, Department of Endocrine and Metabolic Diseases, 20145 Milan, Italy

**Keywords:** β-cell glucose sensitivity, insulin clearance, glucose tolerance

## Abstract

**Background/Objectives**: To investigate how β-cell glucose sensitivity, insulin clearance, and insulin sensitivity interact to determine glucose tolerance in a population without overt diabetes. **Methods**: We analyzed data from 54 individuals without diabetes (age: 44 years, IQR: 27–56; 63% females; BMI: 24.5 kg/m^2^, IQR: 21.9–28.7; HbA1c 33.26 mmol/mol, IQR: 32.13–35.51) undergoing a 3-h OGTT. β-cell glucose sensitivity, insulin clearance, and insulin sensitivity were assessed via modeling of OGTT data. Their relationship with glucose tolerance was evaluated through linear regression models. **Results**: β-cell glucose sensitivity strongly predicted glucose tolerance during the OGTT (IQR increase effect: −87 mg/dL; 95% CI: −141, −32; *p* = 0.003) but not fasting glucose (*p* = 0.4). Patients with lower β-cell glucose sensitivity showed the widest range of glucose tolerance during the OGTT, some approaching diabetic levels whereas others tolerating glucose well; insulin sensitivity was the strongest determinant of this variance (IQR increase effect: −49 mg/dL; 95% CI: −68, −31; *p* < 0.001) significantly influencing the relationship between β-cell glucose sensitivity and glucose tolerance (interaction term *p* = 0.035). Conversely, insulin clearance did not show a statistically significant association with mean glucose levels during the OGTT (β: 4.2; 95% CI: −8.0, 16; *p* = 0.5). However, a non-linear relationship between insulin clearance and β-cell glucose sensitivity was identified, and three distinct metabolic subgroups were defined, highlighting the heterogeneity underlying the development of dysglycemia. **Conclusions**: β-cell glucose sensitivity is the primary determinant of glucose tolerance during an oral glucose challenge. While high β-cell glucose sensitivity often overcomes low insulin sensitivity, the latter becomes crucial when β-cell glucose sensitivity is low. The identification of distinct metabolic profiles, related to insulin secretion and clearance, highlights the heterogeneity of the transition from glucose tolerance to dysglycemia.

## 1. Introduction

Glucose tolerance is a critical aspect of metabolic health. Impaired glucose tolerance (IGT) and impaired fasting glucose (IFG) are both indicators of prediabetes, a significant risk factor for progression to type 2 diabetes and cardiovascular disease [1].

The numerous determinants of glucose tolerance include both insulin secretion and the responsiveness of various tissues and organs to insulin signaling.

Impaired β-cell function is a key element in the pathogenesis of dysglycemia. Indeed, impairment in β-cell function occurs not only in type 1 diabetes, which has an autoimmune origin, but also in type 2 diabetes. In some cases of dysglycemia, evidence indicates that β-cell dysfunction appears early, and it is already present during the initial stages of IGT [2]. Thus, reduced β-cell secretion may precede overt hyperglycemia, indicating that loss of β-cell function is an early, critical event in the natural history of prediabetes and type 2 diabetes [2,3].

More specifically, individuals who later develop dysglycemia already exhibit lower β-cell glucose sensitivity, defined as the capacity of β cells to enhance insulin secretion in response to rising blood glucose levels, even when normoglycemic at baseline [4].

A key factor in the progression from normoglycemia to IGT is whole-body insulin sensitivity, which reflects how efficiently peripheral tissues dispose of glucose in response to insulin.

Reduced insulin sensitivity at baseline in normoglycemic individuals is an independent predictor of progression to hyperglycemia. In other words, decreased insulin sensitivity precedes the deterioration of glucose homeostasis, marking, together with reduced β-cell glucose sensitivity, an early pathogenic step before overt impairment of glucose tolerance [4].

Reduced β-cell glucose sensitivity correlates with elevated 2-h plasma glucose levels during an oral glucose tolerance test (OGTT), and concurrent low insulin sensitivity further exacerbates these glucose excursions [5].

Conversely, adequate insulin sensitivity can maintain euglycemia even when β-cell glucose sensitivity is modestly reduced. Thus, preserved insulin sensitivity compensates for mild deficits in β-cell glucose responsiveness [5].

Another fundamental aspect of glucose metabolism, whose role in the development of glucose tolerance impairment is still unclear, is insulin clearance [6].

Insulin clearance reflects the rate at which circulating insulin is removed from the bloodstream, primarily via hepatic first-pass extraction and, to a lesser extent, via extra-hepatic degradation [6].

At standardized insulin secretion rates, insulin clearance correlates positively with whole-body insulin sensitivity. When the latter is reduced, insulin clearance decreases as an adaptive response aimed at preserving higher circulating insulin concentrations and, therefore, euglycemia, despite insulin resistance [7].

In general, as insulin levels rise, clearance declines due to a saturation effect of the clearance pathways. During an OGTT, this phenomenon becomes evident, helping to sustain higher insulin levels in the postprandial state [8].

OGTT-based modeling approaches have provided key insights into the relationship between β-cell glucose sensitivity and insulin sensitivity in the regulation of glucose tolerance [4,5].

More recently, the additional contribution of insulin clearance to glucose tolerance has been highlighted through extensions of the disposition index framework incorporating insulin clearance [9].

However, insulin clearance has been less consistently integrated into OGTT-based models to determine glucose tolerance. Specifically, OGTT-based modeling approaches simultaneously evaluating β-cell glucose sensitivity, insulin clearance and insulin sensitivity in non-diabetic individuals from the general population remain limited [10,11].

In this context, the aim of the present study was to investigate the interplay between β-cell glucose sensitivity, insulin sensitivity, and insulin clearance in individuals without overt diabetes. We characterized and compared individuals with good versus poor glucose tolerance based on their OGTT results, with a specific focus on the role of β-cell glucose sensitivity and insulin clearance. We hypothesized that individuals with poorer glucose tolerance exhibit impaired β-cell glucose sensitivity and reduced insulin sensitivity, and that insulin clearance represents an additional determinant of glucose tolerance, modulating the relationship between β-cell function and glucose homeostasis.

## 2. Materials and Methods

### 2.1. Study Design, Setting and Participants

The study is a cross-sectional analysis correlating β-cell glucose sensitivity, insulin clearance, and insulin sensitivity, assessed via OGTT modeling.

The study was conducted between May and December 2024 at the International Center for the Assessment of Nutritional Status, a university-affiliated research center specialized in the nutritional management of obesity and its comorbidities.

The inclusion criterion was age between 18 and 65 years.

Exclusion criteria included overt type 2 diabetes, end-stage chronic kidney disease, pregnancy, breastfeeding, and treatment with hypoglycemic agents other than metformin.

A total of 54 individuals met the eligibility criteria and were therefore enrolled, all on a voluntary basis. All assessments were performed in the same morning for each participant.

### 2.2. Variables and Measurements

The main study outcomes were β-cell glucose sensitivity, insulin clearance, and insulin sensitivity, which were derived from mathematical modeling of modified OGTT data.

A modified version of the OGTT was performed: after the oral administration of 75 g of glucose, blood samples were collected every 30 min from 0 to 120 min and then at 180 min to measure plasma glucose, insulin and C-peptide concentrations.

These parameters were used to estimate the aforementioned outcomes by applying a model describing the relationship between insulin secretion and blood glucose concentrations [12].

The model expresses insulin secretion during an OGTT as the sum of distinct components. The primary component is a dose–response function describing the relationship between glucose concentration and insulin secretion. The slope of this function represents β-cell glucose sensitivity, reflecting the ability of β-cells to increase insulin secretion in response to rising glucose levels.

The second component is a derivative component that reflects rate sensitivity, i.e., the early insulin response to a rapid increase in glucose concentrations. This component is proportional to the rate of change in glucose levels.

The third component is a potentiation factor, which modulates the dose response relationship over time, accounting for slower regulatory mechanisms such as incretin effects. A full mathematical description of the model has been previously reported [12].

All parameters were assayed by a commercial kit (Roche Diagnostics, Basel, Switzerland) with Cobas Integra 400 Plus and Cobas 411 (Roche Diagnostics, Basel, Switzerland).

Anthropometric measurements included weight and height, used to calculate body mass index (BMI), waist and hip circumference, which were used to calculate waist-to-hip ratio (WHR), skinfold thick-ness at biceps, triceps, subscapular and suprailiac sites to estimate fat mass percentage.

Abdominal ultrasounds were performed on fasting subjects by the same operator. Visceral adipose tissue and subcutaneous adipose tissue were measured 1 cm above the umbilicus. The examination was performed at end-expiration, applying the same probe pressure for all subjects. Subcutaneous adipose tissue was measured with the 7.5 MHz linear probe as the distance between the epidermis and the external face of the rectus abdominis muscle. Visceral adipose tissue was measured with the 3.5 MHz convex-array probe as the distance between the anterior wall of the aorta and the posterior surface of the rectus abdominis muscle.

Blood pressure was also recorded.

A structured medical interview was conducted to collect information about family history on obesity, diabetes, cardiovascular and cerebrovascular disease, as well as personal medical history and physical activity levels.

Dietary habits and eating patterns were analyzed using validated food-frequencies questionnaires (FFQ) [13,14,15,16,17].

All measurements were performed by medical doctors and registered dietitians using standardized procedures to minimize measurement errors [18,19,20].

### 2.3. Study Size and Statistical Methods

Participant characteristics were summarized and presented stratified by sex.

Continuous variables, which were not normally distributed, were presented as medians and interquartile ranges (IQRs; 25th, 75th percentile). Categorical variables were presented as counts (n) and percentages (%).

To investigate the interplay of metabolic factors in determining glucose tolerance, three separate multiple linear regression models were fitted. The dependent variables for these models were (1) Basal Glucose, (2) Glucose at 2 h, and (3) Mean Glucose during OGTT. Predictors for the “Basal Glucose” model were β-cell glucose sensitivity, Basal Insulin Clearance Rate, and an insulin resistance index. The models for “Glucose at 2 h” and “Mean Glucose during OGTT” used β-cell glucose sensitivity, Mean Insulin Clearance during OGTT, and the Oral Glucose Insulin Sensitivity (OGIS) at 2 h as predictors. All models included main effects for the three key predictors as well as two interaction terms to test for effect modification: (β-cell glucose sensitivity × Insulin Clearance) and (β-cell glucose sensitivity × Insulin Sensitivity). Predictors were scaled by their IQR, so their coefficients (Beta) represent the change in the outcome variable for an IQR increase in the predictor.

An a priori sample size calculation was performed to ensure the development of a robust predictive model for “Mean Glucose during OGTT.” The planned model included 5 main predictors and interaction terms. Based on prior literature indicating strong relationships between insulin sensitivity, secretion, and glucose tolerance, we anticipated a model R^2^ of at least 0.60. To ensure model stability and limit overfitting (targeting a relative shrinkage of < 10%), a minimum sample size of N = 50 was determined to be sufficient. At this sample size, an anticipated R^2^ = 60 with 5 predictors would yield an expected shrinkage of approximately 7%, which is well below the 10% threshold.

To define a clinically relevant threshold for β-cell glucose sensitivity, a Receiver Operating Characteristic (ROC) curve analysis was performed. The clinical outcome used as the reference was glucose tolerance status. Individuals were categorized as having ‘Normal’ glucose tolerance (fasting glucose < 100 mg/dL and 2-h glucose < 140 mg/dL) or ‘Worse’ pre-diabetic tolerance (fasting glucose ≥ 100 mg/dL or 2-h glucose ≥ 140 mg/dL, consistent with IFG and/or IGT). To ensure robust interpretation and minimize bias, age-, sex-, BMI-, and physical activity-adjusted differences in metabolic characteristics between these two groups were assessed using Analysis of Covariance (ANCOVA).

All statistical analyses were performed using R 4.5.2. A *p*-value of < 0.05 was considered statistically significant.

## 3. Results

The study included 54 individuals without diabetes. The median age was 44 years (IQR: 27–56), and 63% (N = 34) were female. Participants had a median BMI of 24.5 kg/m^2^ (IQR: 21.9–28.7) and a median HbA1c of 33.26 mmol/mol (IQR: 32.13–35.51). While our exclusion criteria permitted the use of metformin, no participants enrolled in the final study cohort were actively undergoing treatment with any hypoglycemic agents. Detailed baseline characteristics, including glycemic status categories, comorbidities, and family history, are presented in Table 1.

Median values for the key metabolic parameters, derived from the OGTT modeling, are shown in Table 2. Overall, the median glucose during the OGTT was 108 (IQR: 91, 126) mg/dL, β-cell glucose sensitivity was 121 (IQR: 74–172) pmol × min^−1^ × m^−2^ × mmol^−1^ × L, the basal insulin clearance rate was 1.60 (IQR: 1.26–2.14) L/min/m^2^, and the OGIS at 2 h was 413 (IQR: 352–454) mL/min/m^2^.

The linear multivariate regression models (Table 3) revealed that β-cell glucose sensitivity was a strong predictor of glucose tolerance after a glucose challenge, but it didn’t have an independent effect for fasting glucose (*p* > 0.05). An IQR increase in β-cell glucose sensitivity was associated with an 87 mg/dL lower mean glucose concentration during the OGTT (95% CI: −141, −32; *p* = 0.003) and a 97 mg/dL lower glucose concentration at 2 h (*p* = 0.012). However, it had no significant effect on fasting glucose (*p* = 0.4).

Insulin sensitivity was also a strong predictor across all glucose measures (*p* < 0.001 for all). Conversely, insulin clearance was not a major determinant of glucose regulation, and its effect on mean OGTT glucose was minor and not statistically significant (Beta: 4.2; 95% CI: −8.0, 16; *p* = 0.5).

**Figure 1:**
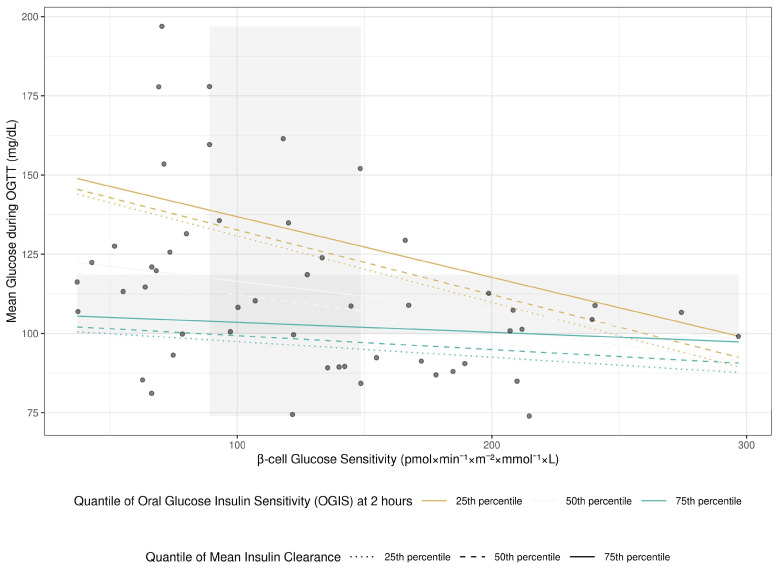
Relationship between Mean Glucose during OGTT and Beta-cell Glucose Sensitivity, Adjusted for Insulin Clearance and Sensitivity. Points represent real data; lines represent fitted values from the model.

A significant interaction was found between β-cell glucose sensitivity and insulin sensitivity in predicting mean OGTT glucose (interaction term *p* = 0.035). This interaction is visualized in Figure 1. Individuals with high β-cell glucose sensitivity (e.g., >200) managed glucose levels well, with mean OGTT glucose remaining relatively low, irrespective of insulin sensitivity status. However, in individuals with low β-cell glucose sensitivity, insulin sensitivity became a critical determinant of glucose tolerance. This group exhibited the widest range of glucose tolerance, with those who also had low insulin sensitivity (e.g., 25th percentile OGIS) showing the poorest glucose control.

The final model for “Mean Glucose during OGTT” (N = 52, *p* = 5) achieved a high explanatory power (R^2^ = 0.726) with an adjusted R^2^ of 0.696. The calculated relative shrinkage was only 4.1%, confirming the a priori assumption that the sample size was sufficient to prevent model overfitting.

We further explored the relationship between β-cell glucose sensitivity and glucose tolerance using ROC analysis (Supplementary Figure S1). A threshold of β-cell glucose sensitivity was identified at 134 pmol × min^−1^ × m^−2^ × mmol^−1^ × L, below which, individuals were at increased risk of developing IGT or IFG. The model yielded an Area Under the Curve (AUC) of 0.67 (95% CI: 0.51–0.82), with a sensitivity of 0.81 (95% CI: 0.5–1) and a specificity of 0.57 (95% CI: 0.4–0.89).

**Figure 2:**
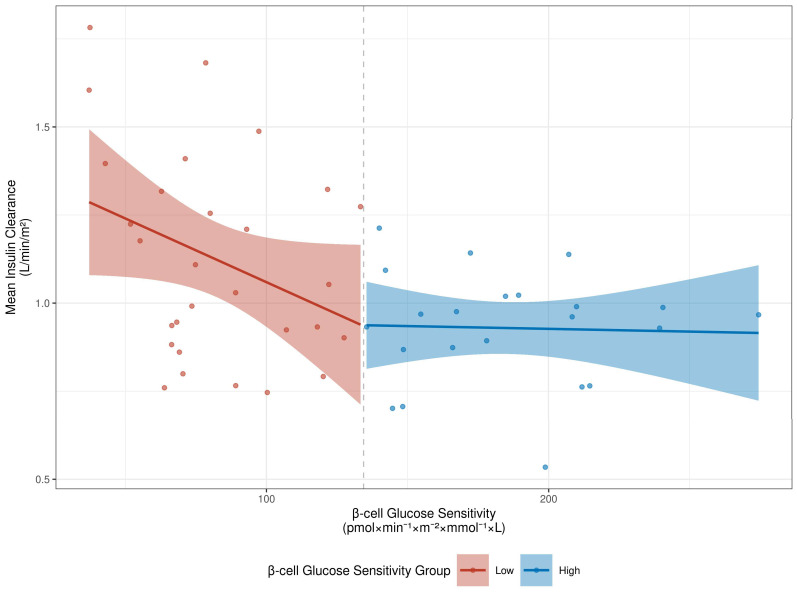
Relationship between β-cell Glucose Sensitivity and Mean Insulin Clearance, Stratified by the ROC-derived Threshold.

Figure 2 illustrates the observed relationship between β-cell glucose sensitivity and mean insulin clearance, stratified by the ROC-derived β-cell glucose sensitivity threshold. We observed a non-linear inverse relationship between β-cell glucose sensitivity and insulin clearance. Clearance rates were higher in individuals with low β-cell sensitivity, while among individuals with preserved β-cell function, clearance rates plateaued and showed no significant association with secretion parameters.

Differences between patients stratified by β-cell glucose sensitivity (low vs. high, defined by the ROC-derived threshold) were assessed after comprehensive adjustment for age, sex, BMI, and physical activity (Supplementary Table S1). Even after these adjustments, mean glucose levels during the OGTT remained significantly higher in low secretors compared to high secretors (120 vs. 97 mg/dL, adjusted difference: 13 mg/dL [95% CI: 2.1, 25], *p* = 0.021, Cohen’s d = 0.951). This large effect size (d > 0.8) and the strictly positive confidence interval indicate a robust, clinically meaningful deterioration in glucose handling associated with poor β-cell sensitivity. Furthermore, individuals with higher β-cell glucose sensitivity continued to exhibit greater insulin secretion (mean insulin during the OGTT: 356 vs. 259 pmol/L, adjusted difference: 144 pmol/L [95% CI: 34, 255], *p* = 0.012, Cohen’s d = 0.485) and lower insulin clearance (mean insulin clearance during the OGTT: 0.96 vs. 1.05 L/min/m^2^, adjusted difference: −0.24 L/min/m^2^ [95% CI: −0.39, −0.10], *p* = 0.002, Cohen’s d = 0.794).

Focusing on the subgroup with low β-cell glucose sensitivity (Supplementary Table S2), those with normal glucose tolerance were characterized by significantly higher oral glucose insulin sensitivity (OGIS: 434 vs. 346, adjusted difference: 54 mL/min/m^2^ [95% CI: 17, 90], *p* = 0.006, Cohen’s d = 1.64). This very large effect size (d > 1.5) and the wide confidence interval emphasize that within this compromised β-cell phenotype, insulin sensitivity acts as the primary and most powerful differentiator of clinical outcome. Additionally, the normal tolerance group exhibited a trend toward lower overall insulin secretion during the OGTT (200 vs. 393 pmol/L, *p* = 0.12, Cohen’s d = 1.07) and maintained significantly higher insulin clearance rates (1.25 vs. 0.94 L/min/m^2^, adjusted difference: 0.27 L/min/m^2^ [95% CI: 0.02, 0.53], *p* = 0.035, Cohen’s d = 1.11).

**Figure 3:**
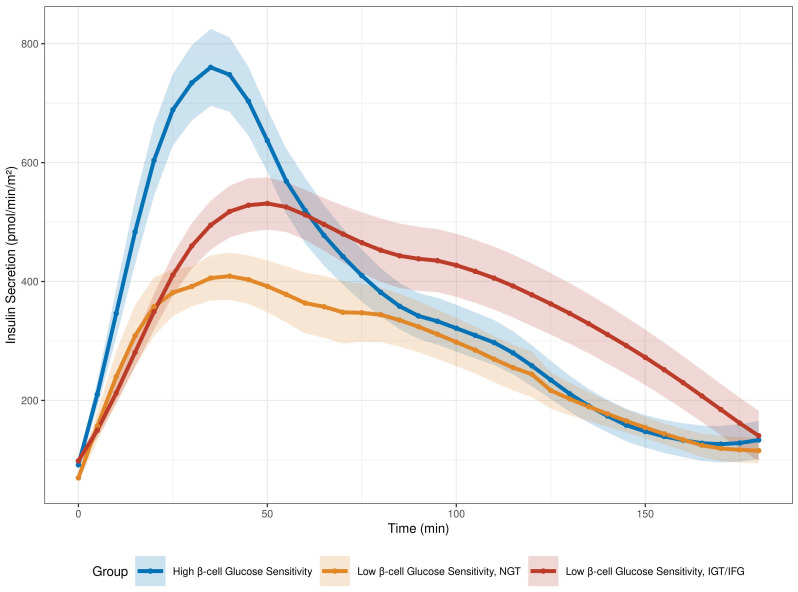
Insulin secretion dynamics during the OGTT across metabolic phenotypes. Curves represent mean insulin secretion rates with 95% confidence intervals (shaded regions). Blue line: individuals with high β-cell glucose sensitivity. Orange line: individuals with low β-cell glucose sensitivity who maintain Normal Glucose Tolerance (NGT) and high oral glucose insulin sensitivity (OGIS). Red line: individuals with low β-cell glucose sensitivity combined with Impaired Glucose Tolerance (IGT) or Impaired Fasting Glucose (IFG), and low OGIS.

Based on these findings, we identified three illustrative phenotypes, whose divergent insulin secretion patterns are illustrated in Figure 3.

The first group consists of individuals with high β-cell glucose sensitivity, characterized by a rapid, high-magnitude secretory peak.

The second group includes individuals with low β-cell glucose sensitivity but high OGIS; despite the blunted early secretory response, these individuals maintain Normal Glucose Tolerance (NGT).

The third group comprises individuals with low β-cell glucose sensitivity and low OGIS; this group corresponds to those with IGT or IFG, who exhibit a delayed and compensatory hypersecretion of insulin in an attempt to overcome resistance.

## 4. Discussion

The present study investigated the complex interplay between β-cell glucose sensitivity, insulin clearance and insulin sensitivity in individuals without overt diabetes. Consistent with established literature, our findings confirm that β-cell glucose sensitivity and insulin sensitivity are the primary determinants of glucose tolerance during an oral challenge [4]

Reduced β-cell glucose sensitivity has been widely recognized as an early pathogenic step in the deterioration of glucose tolerance [2,3]. OGTT-based studies have shown that early alterations in β-cell responsiveness play a central role in the progression toward glucose intolerance [4].

The interaction between β-cell glucose sensitivity and insulin sensitivity in predicting mean OGTT glucose is illustrated in Figure 1. Individuals with high β-cell glucose sensitivity maintained lower mean glucose levels during the OGTT, despite differences in insulin sensitivity status. Conversely, among subjects with low β-cell glucose sensitivity, insulin sensitivity plays a critical role in determining glucose tolerance: when both are impaired, marked deterioration in glycemic control appears.

Insulin clearance represents an important component of glucose homeostasis, as it regulates circulating insulin availability and therefore modulates the balance between insulin secretion, insulin sensitivity, and blood glucose levels [6].

Beyond these established mechanisms, our study provides novel evidence that insulin clearance plays a dynamic role in modulating glucose tolerance. Clearance exhibits a distinct U-shaped relationship with β-cell function, modulating insulin availability in response to the prevailing secretory status.

Although linear regression models suggested a limited role for insulin clearance, further analyses revealed a non-linear nature of the relationship between clearance and β-cell glucose sensitivity, as visualized in Figure 2. Interestingly, the highest insulin clearance rates were observed in individuals with the lowest β-cell glucose sensitivity.

Three illustrative phenotypes emerged from the analyses:

○ High β-cell glucose sensitivity: characterized by a marked early insulin release and large amounts of insulin extracted by the liver over a short time window.
○ Low β-cell glucose sensitivity with high OGIS: individuals who maintain glucose tolerance and show a reduced early insulin response and moderate, yet prolonged, insulin secretion; in this scenario, we hypothesize that insulin clearance is high due to continued hepatic extraction.
○ Low β-cell glucose sensitivity with low OGIS: characterized by impaired glucose tolerance and show a weaker insulin response, followed by prolonged and increased insulin secretion during the OGTT, leading to exposure of the liver to elevated insulin levels and likely leads to a saturation of its extraction capacity, resulting in intermediate mean insulin clearance.

Overall, these results indicate that insulin clearance represents an additional physiological mechanism contributing to inter-individual variability in glucose tolerance and may help define distinct metabolic phenotypes.

These three phenotypes highlight the heterogeneous trajectories toward dysglycemia. The high β-cell glucose sensitivity phenotype relies on a robust, early-phase secretory burst that rapidly suppresses hepatic glucose production, allowing for a quick return to baseline. In contrast, individuals with low β-cell sensitivity but normal tolerance manage to maintain euglycemia not through a burst, but through sustained, moderate insulin secretion. Finally, the IGT/IFG group is characterized by the loss of early-phase secretion that triggers a delayed, compensatory hyperinsulinemia that is ultimately insufficient to overcome resistance, leading to prolonged hyperglycemic exposure.

The identification of distinct metabolic profiles may enable more precise and customized preventive and therapeutic strategies in clinical practice.

This study has potential limitations that should be considered when interpreting the results.

First, the study population was a convenience sample from a single center, which may limit the generalizability of our findings to other settings with different patient demographics and metabolic characteristics, such as BMI distribution, age distribution and baseline glucose tolerance status.

The relatively small sample size (n = 54) may reduce the statistical power to detect subtle associations among the investigated variables.

In addition, the cross-sectional design of the study precludes establishing causal relationships between the investigated variables and glucose tolerance status.

The metabolic diversity of the cohort can be considered both a strength and a limitation. While it may limit the generalizability of the results to other populations, it also highlights the underlying metabolic heterogeneity among normoglycemic individuals and allows for the identification of three distinct metabolic phenotypes.

The primary predictors (β-cell glucose sensitivity, insulin clearance, and insulin sensitivity) were not directly measured using gold-standard clamp techniques but were estimated from mathematical modelling of OGTT data. Consequently, the validity of these estimates is dependent on the accuracy and assumptions of the underlying physiological models. This could attenuate or bias the observed associations.

Additionally, although key metabolic variables were included in the models, residual confounding from unmeasured factors (e.g., diet composition, detailed physical activity levels, or liver fat content) could have influenced the observed relationships.

Another limitation of this study is that glucose effectiveness, which has been established as a significant determinant of glucose homeostasis in both fasting and postprandial state, was not assessed [21,22]. Its omission may have resulted in an incomplete characterization of the mechanisms regulating glucose tolerance and could have influenced the observed relationships between β-cell glucose sensitivity, insulin sensitivity, and insulin clearance.

Furthermore, our study was designed to evaluate β-cell glucose sensitivity as a foundational physiological trait that determines glucose tolerance, rather than investigating it as an outcome. Consequently, our dataset is not equipped to identify the upstream etiologies that are responsible for the reduced β-cell sensitivity observed in these individuals.

## 5. Conclusions

In conclusion, this study underscores the complex, dynamic interplay between β-cell glucose sensitivity, insulin sensitivity, and insulin clearance in maintaining glucose homeostasis among individuals without overt diabetes. Beyond confirming the primary roles of β-cell function and insulin resistance, our findings highlight a non-linear contribution of insulin clearance, culminating in the identification of three distinct metabolic phenotypes. These profiles reveal heterogeneous physiological trajectories toward dysglycemia, demonstrating that individuals with similar glucose tolerance statuses may rely on fundamentally different compensatory mechanisms. Further studies, including larger cohorts and individuals across the full spectrum of glucose tolerance, may help to clarify the relative contribution of insulin clearance in different insulin secretory phenotypes.

## Figures and Tables

**Table 1:** Patient Characteristics, Stratified by Sex.

Characteristic	Overall N = 54 ^1^	Female N = 34 ^1^	Male N = 20 ^1^
Age (years)	44 (27, 56)	48 (26, 57)	33 (28, 56)
Body Mass Index (BMI) (kg/m^2^)	24.5 (21.9, 28.7)	23.5 (20.8, 29.7)	25.0 (24.4, 27.8)
BMI Category			
Underweight	2 (3.7%)	2 (5.9%)	0 (0%)
Normal weight	28 (52%)	19 (56%)	9 (45%)
Overweight	15 (28%)	6 (18%)	9 (45%)
Obesity class I	6 (11%)	5 (15%)	1 (5.0%)
Obesity class II	2 (3.7%)	1 (2.9%)	1 (5.0%)
Obesity class III	1 (1.9%)	1 (2.9%)	0 (0%)
Waist Circumference (cm)	86 (73, 94)	81 (71, 94)	88 (83, 97)
Waist Circumference Category			
Normal	31 (57%)	17 (50%)	14 (70%)
Elevated	7 (13%)	5 (15%)	2 (10%)
Central obesity	16 (30%)	12 (35%)	4 (20%)
Hypertension Diagnosis (Yes/No)	5 (9.3%)	3 (8.8%)	2 (10%)
Cardiovascular Disease Diagnosis (Yes/No)	2 (3.7%)	1 (2.9%)	1 (5.0%)
Dyslipidemia/Hypercholesterolemia Diagnosis (Yes/No)	10 (19%)	8 (24%)	2 (10%)
Family History of Hypertension (Yes/No)	36 (67%)	21 (62%)	15 (75%)
Family History of Diabetes (Yes/No)	28 (52%)	17 (50%)	11 (55%)
Family History of Cardiovascular Disease (Yes/No)	32 (59%)	22 (65%)	10 (50%)
Family History of Obesity/Overweight (Yes/No)	30 (56%)	18 (53%)	12 (60%)
Family History of Dyslipidemia (Yes/No)	3 (5.6%)	3 (8.8%)	0 (0%)
Physically Active (Yes/No)	36 (68%)	22 (67%)	14 (70%)
Smoking Status (Yes/No)			
Never	42 (78%)	29 (85%)	13 (65%)
Ex-smoker	2 (3.7%)	1 (2.9%)	1 (5.0%)
Smoker	10 (19%)	4 (12%)	6 (30%)
Fasting Glucose (mg/dL)	96 (88, 102)	93 (87, 101)	99 (96, 104)
Fasting Glucose Category			
Normal	37 (69%)	25 (74%)	12 (60%)
Impaired Fasting Glucose (IFG)	16 (30%)	8 (24%)	8 (40%)
Diabetes	1 (1.9%)	1 (2.9%)	0 (0%)
Glucose at 120 min (mg/dL)	93 (77, 118)	89 (70, 115)	100 (83, 129)
2-h Glucose Category			
Normal	46 (85%)	29 (85%)	17 (85%)
Impaired Glucose Tolerance (IGT)	7 (13%)	4 (12%)	3 (15%)
Diabetes	1 (1.9%)	1 (2.9%)	0 (0%)
Glycated Hemoglobin (HbA1c) (mmol/mol)	33.26 (32.13, 35.51)	33.25 (31.99, 35.33)	33.94 (32.15, 35.56)
HbA1c Category			
Optimal	50 (93%)	31 (91%)	19 (95%)
Suboptimal	2 (3.7%)	2 (5.9%)	0 (0%)
Prediabetes	2 (3.7%)	1 (2.9%)	1 (5.0%)
Diabetes	0 (0%)	0 (0%)	0 (0%)

^1^ Median (Q1, Q3); n (%).

**Table 2:** Oral Glucose Tolerance Data, Stratified by Sex.

Characteristic	Overall N = 54 ^1^	Female N = 34 ^1^	Male N = 20 ^1^
Basal Glucose (mg/dL)	96 (88, 102)	93 (87, 101)	99 (96, 103)
Mean Glucose during OGTT (mg/dL)	108 (91, 126)	101 (89, 121)	115 (106, 133)
Glucose at 2 h (mg/dL)	92 (77, 118)	88 (70, 115)	100 (83, 128)
Basal Insulin (pmol/L)	47 (36, 81)	44 (33, 86)	58 (37, 72)
Mean Insulin during OGTT (pmol/L)	319 (216, 428)	347 (216, 446)	302 (226, 398)
Basal Insulin Secretion Rate (pmol × min^−1^ × m^−2^)	84 (67, 105)	84 (56, 105)	87 (69, 113)
Total Insulin Secretion during OGTT (nmol × m^−2^)	56 (44, 73)	58 (45, 80)	54 (39, 71)
β-cell Glucose Sensitivity (pmol × min^−1^ × m^−2^ × mmol^−1^ × L)	121 (74, 172)	125 (74, 178)	114 (74, 158)
Oral Glucose Insulin Sensitivity (OGIS) at 2 h	413 (352, 454)	426 (352, 467)	386 (359, 428)
Stumvoll Insulin Sensitivity Index	8.91 (6.54, 10.75)	9.23 (6.95, 10.82)	8.88 (6.36, 9.25)
Matsuda Insulin Sensitivity Index	5.0 (3.1, 7.2)	5.3 (3.1, 7.2)	4.3 (2.8, 6.5)
Basal Insulin Clearance Rate (L/min/m^2^)	1.60 (1.26, 2.14)	1.71 (1.25, 2.14)	1.55 (1.28, 2.21)
Mean Insulin Clearance Rate during OGTT (L/min/m^2^)	0.97 (0.87, 1.21)	0.99 (0.88, 1.14)	0.96 (0.78, 1.22)

^1^ Median (Q1, Q3).

**Table 3:** Linear Regression Models for Fasting and OGTT Glucose Measures.

	Basal Glucose (mg/dL)			Glucose at 2 h (mg/dL)			Mean Glucose During OGTT (mg/dL)		
Characteristic	Beta	95% CI	*p*-Value	Beta	95% CI	*p*-Value	Beta	95% CI	*p*-Value
β-cell glucose sensitivity (IQR increase)	−7.6	−24, 9.0	0.4	−97	−172, −22	0.012	−87	−141, −32	0.003
Basal or Mean Insulin Clearance Rate (IQR increase)	0.00	−7.7, 7.7	>0.9	0.92	−16, 18	>0.9	4.2	−8.0, 16	0.5
Insulin Resistance or Sensitivity Index (IQR increase)	15	6.6, 23	<0.001	−60	−85, −34	<0.001	−49	−68, −31	<0.001
β-cell glucose sensitivity (IQR increase) × Basal or Mean Insulin Clearance Rate (IQR increase)	2.0	−3.4, 7.4	0.5	5.1	−8.2, 18	0.4	1.9	−7.9, 12	0.7
β-cell glucose sensitivity (IQR increase) × Insulin Resistance or Sensitivity Index (IQR increase)	−1.6	−6.8, 3.5	0.5	17	−3.9, 37	0.11	16	1.2, 31	0.035
R^2^	0.608			0.693			0.726		
Adjusted R^2^	0.565			0.660			0.696		
Sigma	6.29			20.7			15.0		
Statistic	14.3			20.8			24.4		
*p*-value	<0.001			<0.001			<0.001		
df	5			5			5		
Log-likelihood	−166			−228			−212		
AIC	347			470			437		
BIC	360			484			451		
Deviance	1822			19,637			10,396		
Residual df	46			46			46		
No. Obs.	52			52			52		

Abbreviation: CI = Confidence Interval.

## Data Availability

The data presented in this study are available on request from the corresponding author.

## Supplementary Materials

The following supporting information can be downloaded at: https://www.mdpi.com/article/10.3390/metabo16040218/s1, Figure S1: ROC Curve Analysis for β-cell Glucose Sensitivity; Table S1: Age- and Sex-Adjusted Differences in Patients Stratified by β-Cell Glucose Sensitivity (Low vs. High, Defined by ROC Threshold); Table S2: Age- and Sex-Adjusted Differences in Patients With Low β-Cell Glucose Sensitivity (Below ROC Threshold), Stratified by Glucose Tolerance Status; Figure S2: Mean Glucose and Insulin Profiles During the OGTT, Stratified by β-cell Glucose Sensitivity (High vs. Low); Figure S3: Correlation Heatmap of Key Metabolic and Anthropometric Variables.

## Abbreviations

The following abbreviations are used in this manuscript:

IGT	Impaired Glucose Tolerance
IFG	Impaired Fasting Glucose
NGT	Normal Glucose Tolerance
OGTT	Oral Glucose Tolerance Test
OGIS	Oral Glucose Insulin Sensitivity
BMI	Body Mass Index
WHR	Waist Hip Ratio
