# Supplementary material for: Determinants of Glucose Tolerance in a Population Without Overt Diabetes: The Role of β-Cell Glucose Sensitivity, Insulin Sensitivity, and Insulin Clearance
Source: Metabolites. 2026 Mar 26;16(4):218. doi: 10.3390/metabo16040218 (PMC13117718; doi:10.3390/metabo16040218)
Supplement: Supplementary file 1 [file metabolites-16-00218-s001.zip › metabolites-4201392-supplementary.pdf]

Supplementary Material

Figure S1

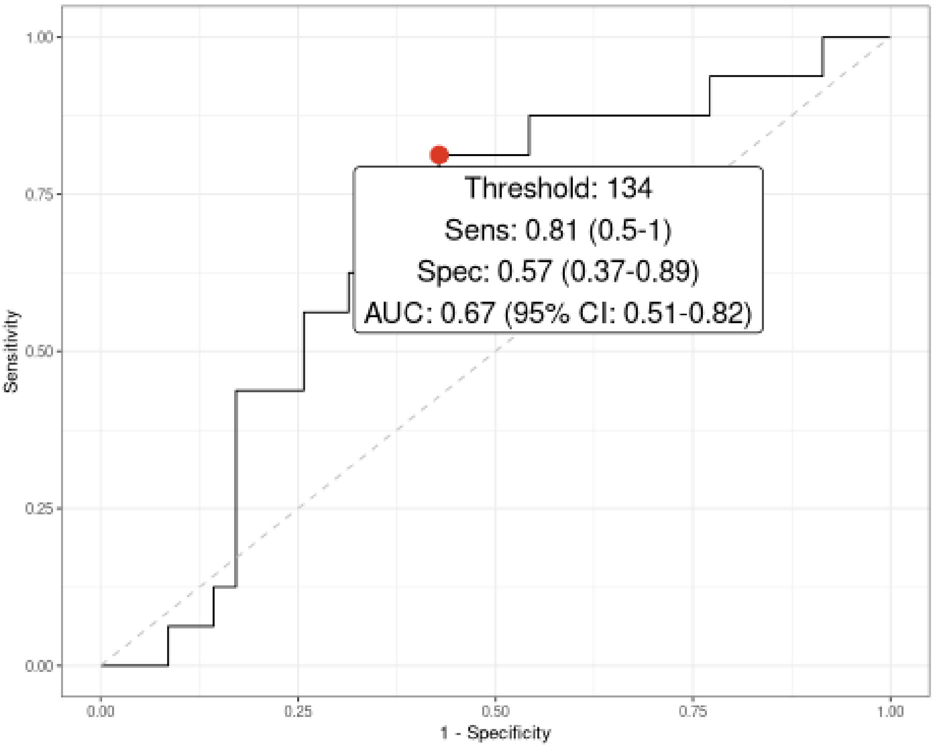

Figure S1: ROC Curve Analysis for β-cell Glucose Sensitivity. The optimal threshold was determined using Youden's J statistic.

Table S1

Table S1: Age-, Sex-, BMI-, and Physical Activity-Adjusted Differences of Patients Stratified by β-Cell Glucose Sensitivity (Low vs. High, Defined by ROC Threshold)

**Characteristic**	**Low** N = 29[1]	**High** N = 23[1]	**Adjusted Difference**[2]	**95% CI**[2]	**p-value**[2]	**Cohen's d**
Waist Circumference (cm)	88 (79, 98)	81 (72, 90)	0.27	-2.3, 2.9	0.8	0.578
Fasting Glucose (mg/dL)	99 (93, 104)	92 (87, 98)	2.5	-2.1, 7.1	0.3	0.639
Glucose at 120 minutes (mg/dL)	105 (82, 129)	83 (70, 99)	11	-4.6, 27	0.2	0.721
Glycated Hemoglobin (HbA1c) (mmol/mol)	34.37 (32.71, 35.54)	32.70 (32.13, 35.18)	0.40	-1.0, 1.8	0.6	0.472
Basal Glucose (mg/dL)	99 (93, 104)	92 (87, 98)	2.5	-2.1, 7.1	0.3	0.639
Mean Glucose during OGTT (mg/dL)	120 (107, 135)	97 (89, 109)	13	2.1, 25	**0.021**	0.951
Glucose at 2 hours (mg/dL)	105 (82, 129)	83 (70, 99)	11	-4.6, 27	0.2	0.721
Basal Insulin (pmol/L)	45 (31, 75)	49 (38, 81)	-12	-28, 3.6	0.12	-0.196
Mean Insulin during OGTT (pmol/L)	259 (188, 416)	356 (305, 428)	-144	-255, -34	**0.012**	-0.485
Basal Insulin Secretion Rate (pmol×min^{-1}×m^{-2})	79 (60, 105)	84 (70, 105)	-13	-29, 2.2	0.090	-0.224
Total Insulin Secretion during OGTT (nmol×m^{-2})	53 (42, 70)	60 (50, 76)	-13	-25, -0.52	**0.042**	-0.310
β-cell Glucose Sensitivity (pmol×min^{-1}×m^{-2}×mmol^{-1}×L)	75 (66, 100)	181 (149, 210)	-105	-124, -85	**<0.001**	-3.19
Rate Sensitivity Index (pmol×m^{-2}×mmol^{-1}×L)	614 (382, 931)	800 (93, 2,199)	-669	-1,532, 194	0.13	-0.500
Insulin Secretion at 5.5 mmol/L Glucose (pmol×min^{-1}×m^{-2})	221 (138, 256)	304 (251, 356)	-91	-149, -33	**0.003**	-1.06
Insulin Secretion at 6.8 mmol/L Glucose (pmol×min^{-1}×m^{-2})	330 (207, 389)	588 (507, 629)	-252	-318, -187	**<0.001**	-2.33
Adjusted Insulin Secretion at 5.5 mmol/L Glucose (pmol×min^{-1}×m^{-2})	82 (66, 103)	107 (82, 143)	-36	-63, -7.8	**0.013**	-0.811
Adjusted Insulin Secretion at 6.8 mmol/L Glucose (pmol×min^{-1}×m^{-2})	126 (108, 168)	229 (153, 284)	-131	-191, -71	**<0.001**	-1.16
Ratio between the mean potentiation factor values in the intervals [100-120] and [0-20] min	2.23 (1.78, 2.61)	2.32 (1.51, 2.65)	-0.20	-0.81, 0.40	0.5	-0.312

Ratio between the mean potentiation factor values in the intervals [160-180] and [0-20] min	1.88 (1.25, 2.48)	1.82 (1.33, 1.99)	0.17	-0.22, 0.56	0.4	0.103
Oral Glucose Insulin Sensitivity (OGIS) at 2 hours	383 (344, 434)	430 (383, 448)	8.0	-22, 38	0.6	-0.358
Stumvoll Insulin Sensitivity Index	8.86 (6.18, 10.14)	9.20 (7.88, 11.12)	0.09	-0.87, 1.1	0.9	-0.457
Matsuda Insulin Sensitivity Index	5.14 (2.65, 8.03)	4.60 (3.59, 5.94)	2.0	0.50, 3.6	**0.011**	0.312
Basal Insulin Clearance Rate (L/min/m²)	1.51 (1.28, 2.12)	1.64 (1.20, 1.94)	0.18	-0.16, 0.53	0.3	0.099
Mean Insulin Clearance Rate during OGTT (L/min/m²)	1.05 (0.90, 1.32)	0.96 (0.87, 1.02)	0.24	0.10, 0.39	**0.002**	0.794

[1]Median (Q1, Q3)

[2]ANCOVA

Abbreviation: CI = Confidence Interval

Table S2

Table S2: Age-, Sex-, BMI-, and Physical Activity-Adjusted Differences of Patients With Low β-Cell Glucose Sensitivity (Below ROC Threshold), Stratified by Glucose Tolerance Status

**Characteristic**	**Normal** N = 15[1]	**Worse** N = 13[1]	**Adjusted Difference**[2]	**95% CI**[2]	**p-value**[2]	**Cohen's d**
Waist Circumference (cm)	84 (73, 95)	92 (88, 104)	1.5	-2.4, 5.4	0.4	-0.582
Fasting Glucose (mg/dL)	93 (86, 97)	104 (101, 108)	-11	-17, -5.4	**<0.001**	-1.98
Glucose at 120 minutes (mg/dL)	86 (74, 111)	125 (105, 152)	-20	-42, 1.4	0.066	-1.29
Glycated Hemoglobin (HbA1c) (mmol/mol)	33.06 (31.28, 34.37)	35.54 (34.62, 36.53)	-2.2	-4.3, -0.07	**0.044**	-1.29
Basal Glucose (mg/dL)	93 (86, 97)	104 (101, 108)	-11	-17, -5.4	**<0.001**	-1.98
Mean Glucose during OGTT (mg/dL)	107 (93, 122)	126 (120, 160)	-17	-32, -2.7	**0.023**	-1.40
Glucose at 2 hours (mg/dL)	86 (74, 111)	125 (105, 152)	-20	-42, 1.4	0.066	-1.29
Basal Insulin (pmol/L)	31 (22, 45)	73 (54, 95)	-26	-46, -5.9	**0.014**	-1.43
Mean Insulin during OGTT (pmol/L)	200 (164, 289)	393 (257, 447)	-101	-231, 29	0.12	-1.07
Basal Insulin Secretion Rate (pmol×min^{-1}×m^{-2})	67 (51, 93)	104 (68, 124)	-21	-43, 1.4	0.064	-1.08
Total Insulin Secretion during OGTT (nmol×m^{-2})	44 (36, 53)	69 (53, 73)	-11	-29, 6.6	0.2	-0.969
β-cell Glucose Sensitivity (pmol×min^{-1}×m^{-2}×mmol^{-1}×L)	78 (55, 107)	74 (68, 100)	-5.3	-31, 21	0.7	-0.231
Rate Sensitivity Index (pmol×m^{-2}×mmol^{-1}×L)	888 (598, 2,216)	455 (323, 716)	495	-185, 1,175	0.14	0.916
Insulin Secretion at 5.5 mmol/L Glucose (pmol×min^{-1}×m^{-2})	233 (147, 276)	170 (102, 255)	27	-36, 90	0.4	0.616
Insulin Secretion at 6.8 mmol/L Glucose (pmol×min^{-1}×m^{-2})	333 (222, 420)	266 (187, 389)	28	-59, 115	0.5	0.486
Adjusted Insulin Secretion at 5.5 mmol/L Glucose (pmol×min^{-1}×m^{-2})	83 (69, 100)	81 (53, 103)	-0.25	-29, 28	>0.9	-0.017
Adjusted Insulin Secretion at 6.8 mmol/L Glucose (pmol×min^{-1}×m^{-2})	117 (98, 168)	126 (111, 161)	-1.8	-49, 45	>0.9	-0.214

Ratio between the mean potentiation factor values in the intervals [100-120] and [0-20] min	2.50 (2.01, 2.87)	1.98 (1.51, 2.23)	0.18	-0.36, 0.72	0.5	0.514
Ratio between the mean potentiation factor values in the intervals [160-180] and [0-20] min	2.04 (1.38, 2.51)	1.53 (1.20, 2.07)	0.31	-0.37, 0.98	0.3	0.478
Oral Glucose Insulin Sensitivity (OGIS) at 2 hours	434 (383, 492)	346 (319, 371)	54	17, 90	**0.006**	1.64
Stumvoll Insulin Sensitivity Index	9.27 (7.70, 10.77)	6.40 (4.79, 8.89)	0.88	-0.46, 2.2	0.2	1.11
Matsuda Insulin Sensitivity Index	8.03 (5.28, 11.27)	2.81 (2.52, 4.54)	3.2	0.81, 5.6	**0.011**	1.57
Basal Insulin Clearance Rate (L/min/m²)	2.09 (1.28, 2.28)	1.33 (1.27, 1.54)	0.40	-0.12, 0.91	0.12	0.960
Mean Insulin Clearance Rate during OGTT (L/min/m²)	1.25 (1.05, 1.49)	0.94 (0.86, 1.03)	0.27	0.02, 0.53	**0.035**	1.11

[1]Median (Q1, Q3)

[2]ANCOVA

Abbreviation: CI = Confidence Interval

Figure S2

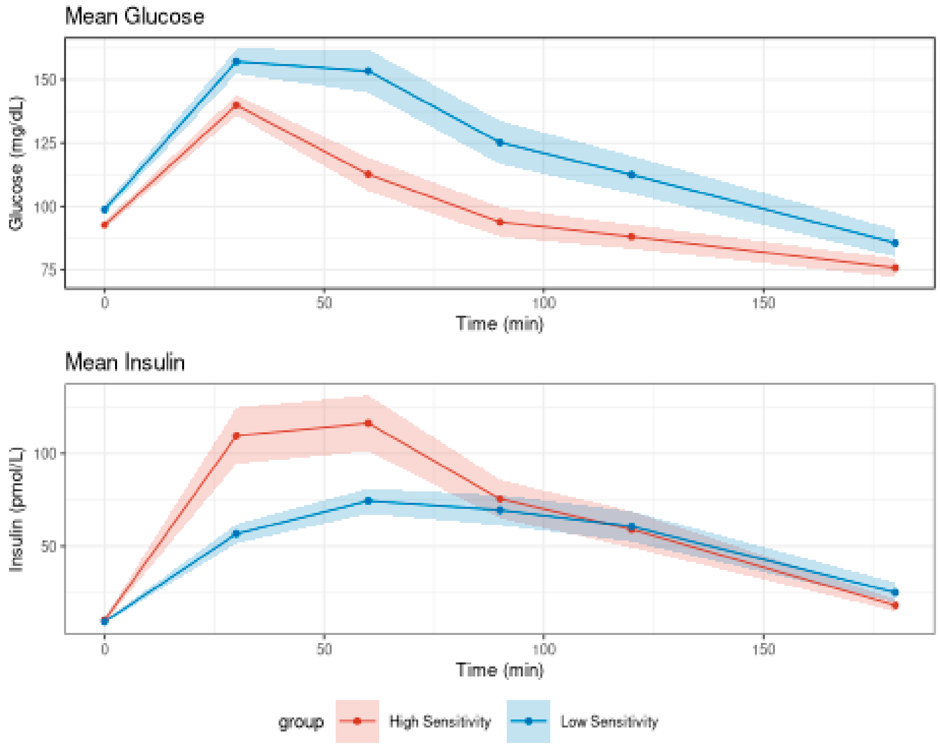

Figure S2: Mean Glucose and Insulin Profiles During the OGTT, Stratified by β-cell Glucose Sensitivity (High vs. Low).

Figure S3

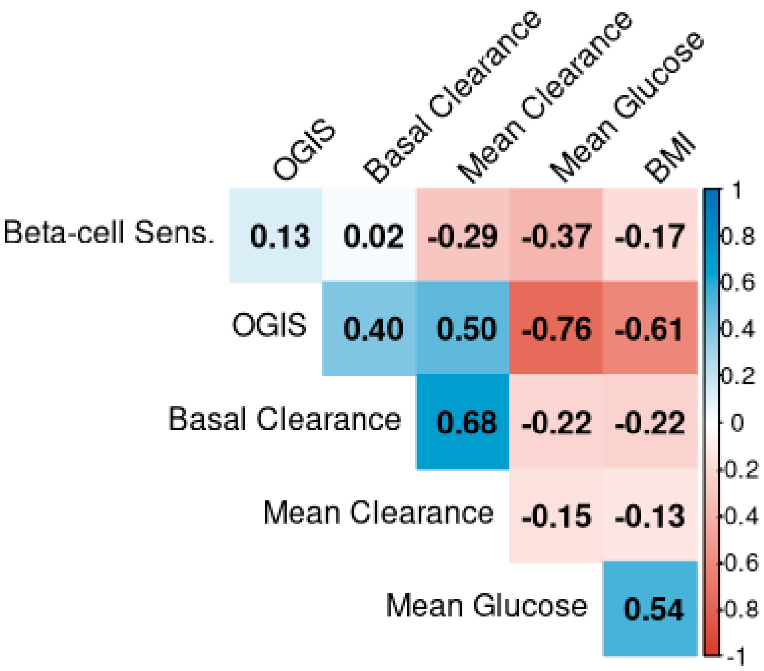

Figure S3: Correlation Heatmap of Key Metabolic and Anthropometric Variables.
